# Chemical Modifications of mRNA Ends for Therapeutic Applications

**DOI:** 10.1021/acs.accounts.3c00442

**Published:** 2023-10-02

**Authors:** Marcin Warminski, Adam Mamot, Anaïs Depaix, Joanna Kowalska, Jacek Jemielity

**Affiliations:** † Division of Biophysics, Institute of Experimental Physics, Faculty of Physics, University of Warsaw, Pasteura 5, 02-093 Warsaw, Poland; ‡ Centre of New Technologies, University of Warsaw, Banacha 2c, 02-097 Warsaw, Poland

## Abstract

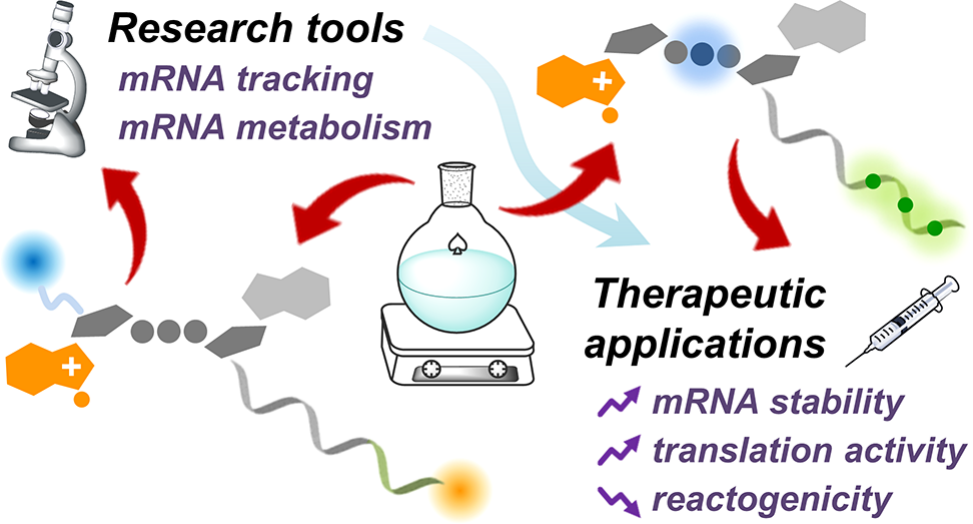

Messenger ribonucleic acid (mRNA) is the universal cellular instruction for ribosomes to produce proteins. Proteins are responsible for most of the functions of living organisms, and their abnormal structure or activity is the cause of many diseases. mRNA, which is expressed in the cytoplasm and, unlike DNA, does not need to be delivered into the nucleus, appears to be an ideal vehicle for pursuing the idea of gene therapy in which genetic information about proteins is introduced into an organism to exert a therapeutic effect. mRNA molecules of any sequence can be synthesized using the same set of reagents in a cell-free system via a process called in vitro transcription (IVT), which is very convenient for therapeutic applications. However, this does not mean that the path from the idea to the first mRNA-based therapeutic was short and easy. It took 30 years of trial and error in the search for solutions that eventually led to the first mRNA vaccines created in record time during the SARS-CoV-2 pandemic. One of the fundamental problems in the development of RNA-based therapeutics is the legendary instability of mRNA, due to the transient nature of this macromolecule. From the chemical point of view, mRNA is a linear biopolymer composed of four types of ribonucleic subunits ranging in length from a few hundred to hundreds of thousands of nucleotides, with unique structures at its ends: a 5′-cap at the 5′-end and a poly(A) tail at the 3′-end. Both are extremely important for the regulation of translation and mRNA durability. These elements are also convenient sites for sequence-independent labeling of mRNA to create probes for enzymatic assays and tracking of the fate of mRNA in cells and living organisms. Synthetic 5′-cap analogs have played an important role in the studies of mRNA metabolism, and some of them have also been shown to significantly improve the translational properties of mRNA or affect mRNA stability and reactogenicity. The most effective of these is used in clinical trials of mRNA-based anticancer vaccines. Interestingly, thanks to the knowledge gained from the biophysical studies of cap-related processes, even relatively large modifications such as fluorescent tags can be attached to the cap structure without significant effects on the biological properties of the mRNA, if properly designed cap analogs are used. This has been exploited in the development of molecular tools (fluorescently labeled mRNAs) to track these macromolecules in complex biological systems, including organisms. These tools are extremely valuable for better understanding of the cellular mechanisms involved in mRNA metabolism but also for designing therapeutic mRNAs with superior properties. Much less is known about the usefulness/utility of poly(A) tail modifications in the therapeutic context, but it is clear that chemical modifications of poly(A) can also affect biochemical properties of mRNA. This Account is devoted to chemical modifications of both the 5′- and 3′-ends of mRNA aimed at improving the biological properties of mRNA, without interfering with its translational function, and is based on the authors’ more than 20 years of experience in this field.

## Key References

- SikorskiP. J.; WarminskiM.; KubackaD.; RatajczakT.; NowisD.; KowalskaJ.; JemielityJ.The identity and methylation status of the first transcribed nucleotide in eukaryotic mRNA 5′ cap modulates protein expression in living cells. Nucleic Acids Res.2020, 48, 1607–16263198442510.1093/nar/gkaa032PMC7038993.^1^ This work shows the effect of the first transcribed nucleotide in trinucleotide cap analogs on the cellular properties of mRNAs.
- WojtczakB. A.; SikorskiP. J.; Fac-DabrowskaK.; NowickaA.; WarminskiM.; KubackaD.; NowakE.; NowotnyM.; KowalskaJ.; JemielityJ.5′-Phosphorothiolate Dinucleotide Cap Analogues: Reagents for Messenger RNA Modification and Potent Small-Molecular Inhibitors of Decapping Enzymes. J. Am. Chem. Soc.2018, 140, 5987–59992967691010.1021/jacs.8b02597.^2^ This work describes one of the most interesting modifications in the cap triphosphate bridge; 5′-phosphorothiolate stabilizes the interaction of the cap structure with eukaryotic translation initiation factor 4E (eIF4E), increases mRNA translation, and significantly reduces the susceptibility of mRNA to decapping.
- WalczakS.; NowickaA.; KubackaD.; FacK.; WanatP.; MroczekS.; KowalskaJ.; JemielityJ.A novel route for preparing 5[prime or minute] cap mimics and capped RNAs: phosphate-modified cap analogues obtained via click chemistry. Chemical Science2017, 8, 260–2672845117310.1039/c6sc02437hPMC5355871.^3^ In this work, it was shown that connecting phosphate residues in the cap structure using click chemistry produces functional mRNAs, which opens the field for capping by click chemistry.
- MamotA.; SikorskiP. J.; SiekierskaA.; de WitteP.; KowalskaJ.; JemielityJ.Ethylenediamine derivatives efficiently react with oxidized RNA 3′ ends providing access to mono and dually labelled RNA probes for enzymatic assays and in vivo translation. Nucleic Acids Res.2022, 50, e33459196410.1093/nar/gkab867PMC8755103.^4^ This work describes how both ends of mRNA can be fluorescently labeled without significant loss of translational properties, creating tools for *in vitro* and *in vivo* mRNA studies.

## 1 Introduction: Therapeutic mRNA—Development, Challenges, Modifications

**Figure 1:**
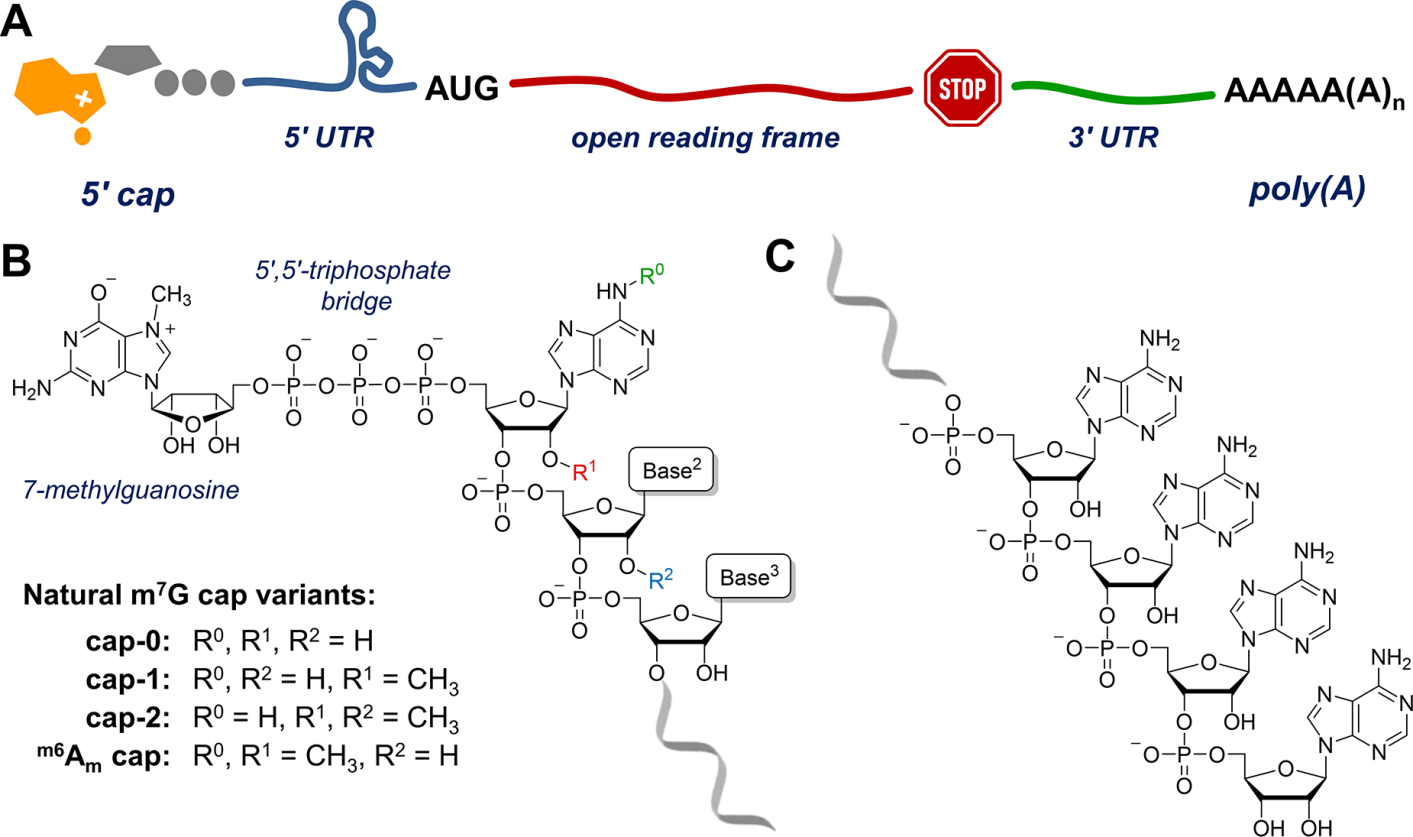
Structure of mRNA: (A) schematic view; (B) mRNA 5′-end (cap); (C) mRNA 3′-end (poly(A) tail).

mRNA is the disposable copy of a particular gene produced during gene expression and serves as a template for protein biosynthesis in the process of mRNA translation. A typical eukaryotic mRNA consists of an open reading frame (protein-coding sequence) flanked by two untranslated regions (5′- and 3′-UTRs) that are specific to the particular gene and two regulatory elements at the very 5′- and 3′-ends that are universal to almost every mRNA (Figure 1A). At the 5′-end, mRNA is capped with an inverted 7-methylguanosine connected to the first nucleotide in the mRNA by a 5′,5′-triphosphate chain (Figure 1B). The 3′-end of mRNA is terminated with a poly(A) tail of a few dozen to ∼250 nucleotides (nt) (Figure 1C). These elements are also essential for high activity of laboratory-produced mRNA designed for therapeutic applications.

The idea of using synthetic mRNA for direct gene transfer in vivo emerged more than 30 years ago but has only recently become a reality beyond laboratory applications with the advent of new-generation vaccines against COVID-19.^5,6^ Using mRNA instead of DNA to deliver genes has several advantages, which stem from the fact that mRNA is disposable and translated in the cytoplasm, and genes delivered in the form of RNA do not need to be integrated with the genome. In the milestone paper, the expression of reporter proteins was observed after direct injection of unmodified mRNA into the skeletal muscles of mice.^7^ Numerous efforts toward advancing mRNA as a therapeutic platform have been made since. Different therapeutic areas, including preventive vaccinations (viral diseases), therapeutic vaccinations (cancer), and protein-replacement therapies were explored.^8^ Methods for improving mRNA properties have also been sought. It turned out to be necessary to apply several “tricks” to ensure mRNA permeability through biological membranes, sufficiently high and prolonged translational activity, and evasion of innate immune responses that have evolved against RNA-based viruses. Therefore, numerous methods have been developed to facilitate delivery, maximize translational activity, increase half-life, and mitigate the reactogenicity of mRNA.^9,10^ The discovery of nonviral delivery based on lipid nanoparticles (LNPs) was a breakthrough step that not only dramatically reduced the doses needed to achieve therapeutic effects but opened the door to the future development of tunable delivery strategies targeting specific tissues and organs.^11,12^ Along with the progress in understanding mRNA biology, strategies for optimizing the sequences of coding and 5′/3′ untranslated regions, and even poly(A) tails of mRNA have emerged.^8,13,14^ Direct chemical modifications of mRNA brought significant advances, as well. Replacing uridine with uridine analogs such as 1-methylpseudouridine in the mRNA body reduced undesired reactogenicity of mRNA.^15,16^ However, the range of chemical modifications applicable to altering the mRNA body is limited, as these modifications must preserve Watson–Crick base-pair interactions and not interfere with ribosome-guided decoding of the open-reading frame. In contrast, the regulatory elements present at the mRNA ends: the 5′-cap and poly(A) tail, offer more space for exploration by bioorganic chemists. Both of these elements can be chemically modified to modulate biological properties of mRNA (especially translational activity) and confer additional features such as the ability to track the mRNA inside cells and/or control activity by external stimuli. Both the 5′-cap and poly(A) tail are universal elements present in almost every eukaryotic mRNA with only a few structural variations. They can be easily incorporated into mRNA either during transcription or post-transcriptionally and are of great importance for the translational activity of mRNA, which is the key feature for therapeutic applications. Therefore, our team has been focusing for many years on the development of chemically modified mRNA cap analogs that may facilitate therapeutic applications of mRNA. Recently, we have also explored modifications of the poly(A) tails to improve stability or enable site-specific labeling of mRNA. We also have shown that combining 5′- and 3′-end modifications gives access to highly pure dual-labeled RNA probes with the potential for future in vivo investigation of mRNA-based drug candidates.

## 2 Why Cap mRNA?

The 7-methylguanosine 5′-cap is a universal modification that marks the 5′-end of almost all eukaryotic mRNAs and fulfills multiple functions crucial for mRNA endurance and translational activity. The key part of the 5′-cap is the inverted guanosine methylated at the *N*7-position and connected to the first nucleotide in the RNA chain by a 5′,5′-triphosphate chain (Figure 1B). This structure, without additional modifications, is known as cap-0. In higher eukaryotes, including humans, the first nucleotides in mRNA are often also 2′-*O*-methylathed to form cap 1 and cap 2 (Figure 1B).

The 5′-cap protects mRNA from 5′-exonucleases and, thereby, prevents premature degradation. Only specialized mRNA decapping enzymes, e.g., Dcp1/Dcp2, can cleave off 7-methylguanosine 5′-diphosphate from mRNA and expose it to rapid exonucleolytic 5′-to-3′ degradation. The 5′-cap is also essential for efficient translation, because it recruits the eukaryotic translation initiation factor 4E (eIF4E). Hence, the 5′-cap is necessary for sufficient stability and efficient translation of mRNA. Furthermore, it has recently come to light that the 5′-cap acts as a specific mark for endogenous mRNAs aiding their differentiation from foreign RNAs, e.g., during viral infection.^17^ Several proteins involved in innate immune response avidly recognize 5′-triphosphate RNAs and even RNAs carrying cap-0, whereas cap-1 RNAs are recognized with much lower affinity.^17^ Recent studies revealed the importance of cap-1 and cap-2 for mammalian development.^18^

The importance of the 5′-cap for mRNA stability, translation, and evasion of the innate immune response requires that any linear mRNA delivered as a therapeutic agent must be capped. In the cell, mRNA capping is realized at an early stage of transcription, by a complex of three enzymatic activities. Due to practical reasons, the capping of in vitro transcribed RNAs is realized by different approaches.

## 3 How to Cap RNA

**Figure 2:**
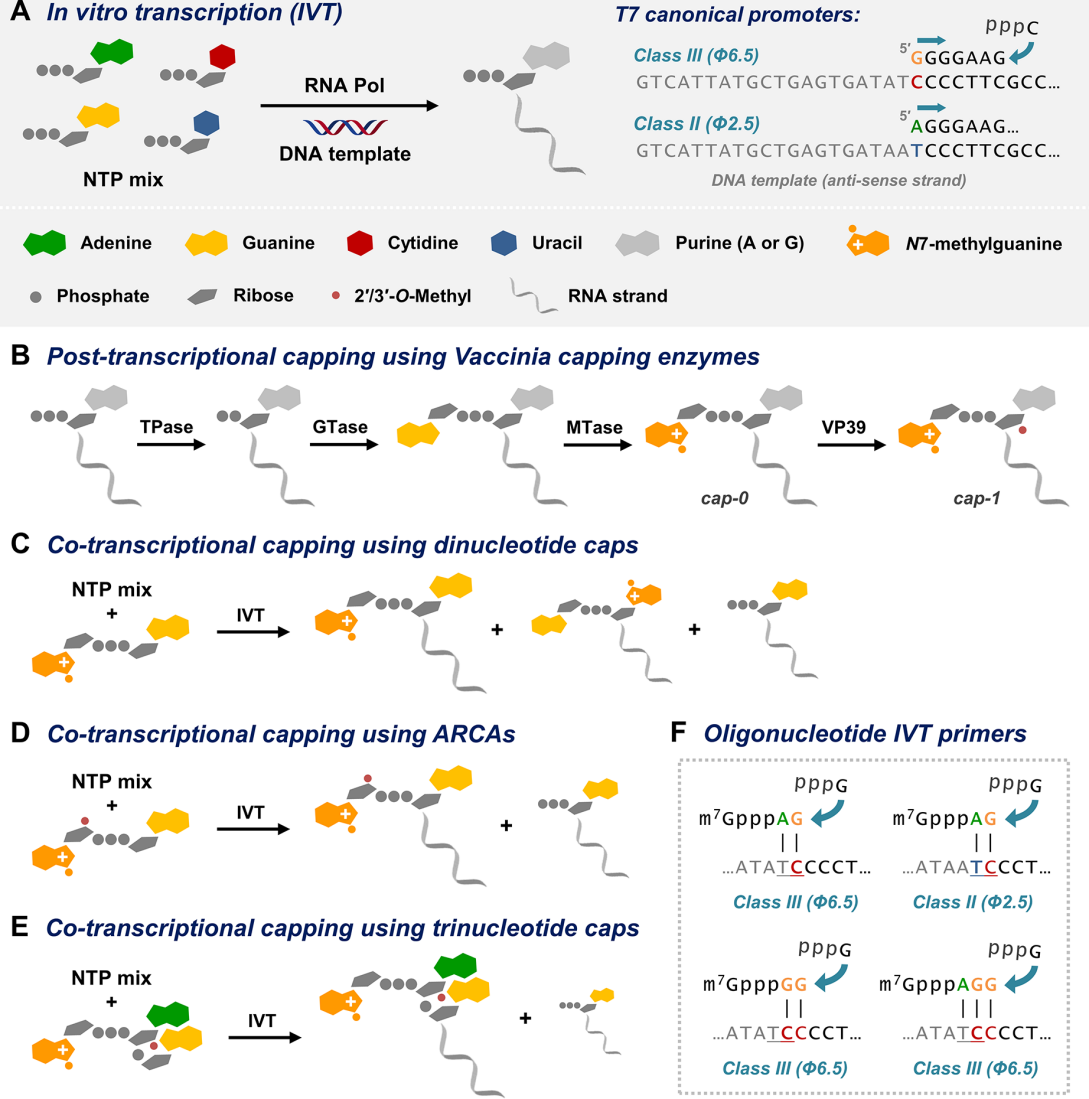
Laboratory methods of mRNA synthesis and capping.

The length of mRNAs is usually far beyond the scope of current chemical synthesis, so their preparation relies on enzymatic methods. In the process called in vitro transcription (IVT), a set of ribonucleoside 5′-triphosphates (NTPs) is polymerized into an RNA chain, according to the nucleotide sequence of the complementary DNA template, in the presence of RNA polymerase (Figure 2A). RNA polymerases of T7, T3, or SP6 bacteriophages, which can initiate the synthesis from a single nucleotide, are typically used to that end. To bind to the template and initiate IVT each polymerase requires a specific sequence, called a promoter site. Transcription from the templates containing T7 class III promoters (e.g., ϕ6.5) produces RNAs starting with guanosine, whereas T7 class II promoters (ϕ2.5) promote the initiation with adenosine.^19^ Initiating RNAs with pyrimidines is more challenging.^20^ During IVT, the oligoribonucleotide chain is elongated in the 5′-to-3′ direction producing 5′-triphosphorylated mRNA. To produce 5′-capped mRNA, additional steps (post-transcriptional capping) or modification of the IVT protocol (co-transcriptional capping) are required.

The process of mRNA capping was first observed in vitro in purified/solubilized viral particles.^21^ Soon after, the protein complex responsible for modifying the mRNA 5′-end was isolated from the vaccinia virus.^22,23^ This so-called Vaccinia Capping Enzyme (VCE) consists of two subunits and combines all three enzymatic activities necessary for adding the 5′-cap on the RNA 5′-triphosphate (Figure 2B). The Vaccinia Capping System (often expanded with 2′-*O*-methyltransferase VP39 from vaccinia to produce cap-1) is commonly used to efficiently cap RNAs, even at a multigram scale.^6,24^

Alternatively, capped RNA can be prepared harnessing the observation that the IVT reaction catalyzed by *E. coli* RNA polymerase and some bacteriophage RNA polymerases (including SP6 and T7) can be primed by 5′,5′-dinucleotides, such as m^7^GpppG (Figure 2C).^25−27^ This method of “co-transcriptional” capping was commonly used to prepare capped RNAs, but only after a decade, Pasquinelli et al. uncovered that about one-third of RNA molecules prepared this way are biologically inactive due to reverse-incorporated m^7^GpppG to form Gppp(m^7^G)-RNA (Figure 2C).^28^

**Figure 3:**
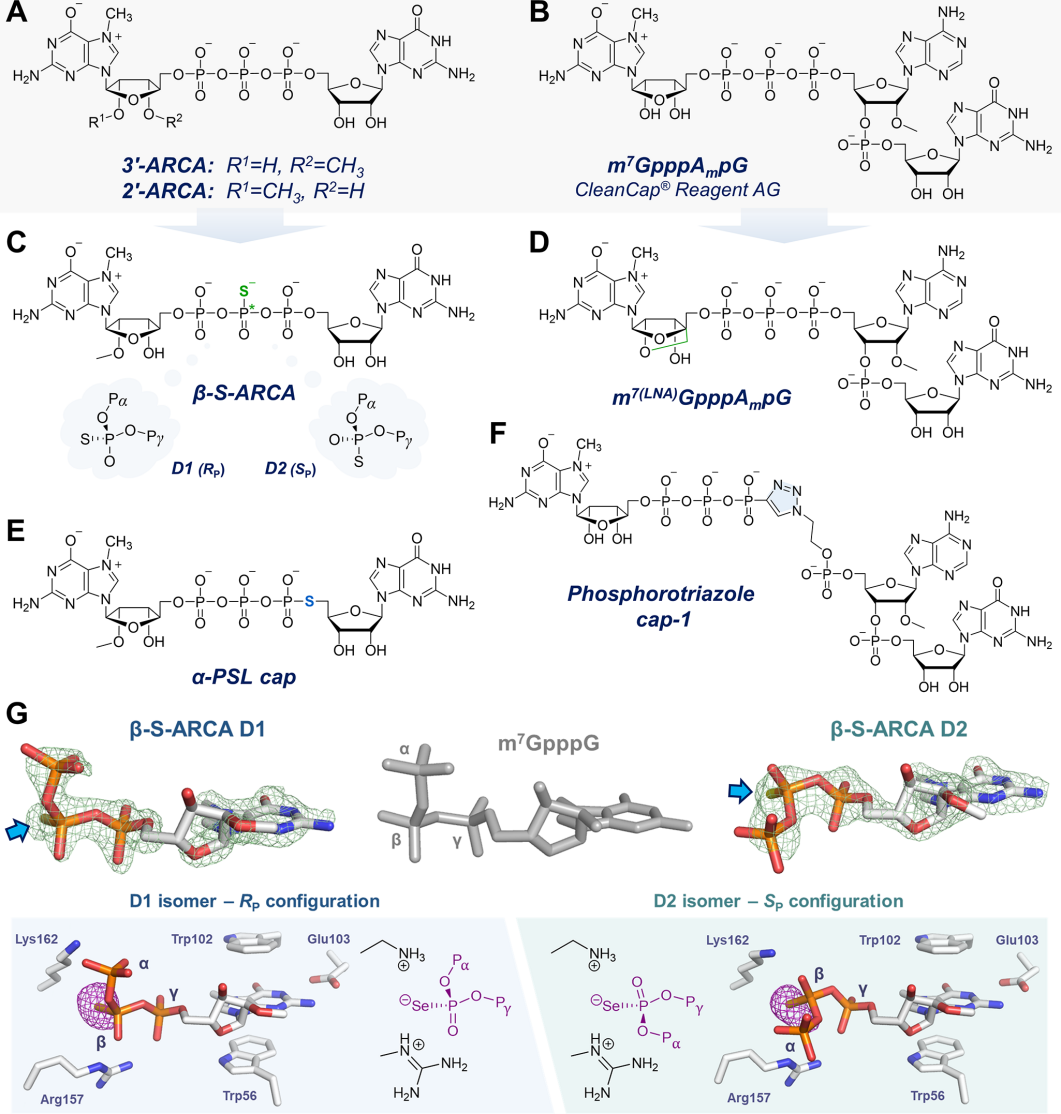
Synthetic cap analogs. (A–F) Chemical structures of selected cap analogs. (G) X-ray structures of β-S-ARCAs in complex with eIF4E (only a part of the cap analogs are visible in the structures); β-sulfur atoms are indicated by blue arrows. Panel G adapted with permission from ref (37). Copyright 2021 American Chemical Society.

The first solution to this problem was “anti-reverse” cap analogs (ARCAs), in which, to prevent RNA polymerase from priming with the m^7^G portion, the 3′-OH group of 7-methylguanosine was either removed (m^7,^3′-dGpppG analog) or methylated (m_2_^7,3′-*O*^GpppG) (Figure 2D).^29^ mRNAs capped with ARCAs were translated in the rabbit reticulocyte lysate over 2-fold more efficiently than mRNA co-transcriptionally capped using m^7^GpppG. A follow-up study revealed similar properties for isomeric ARCA dinucleotides with a 2′-*O*-Me group (Figure 3A).^30^ Although ARCAs offered significant improvement in the synthesis of functional mRNAs, the resulting IVT products still contained a considerable amount (typically 10–50%) of uncapped RNA (pppG-RNA).

The next advance in RNA capping came with the trinucleotide cap analogs. Ishikawa et al. reported a series of m^7^GpppA*pG analogs with differently methylated adenosines (A, A_m_, m^6^A, m^6^A_m_), which acted as efficient IVT primers for T7 RNA polymerase (Figure 2E).^31^ Because the ribose portion of adenosine is not directly involved in the transcription priming, its 2′-*O* position could be methylated to incorporate the cap-1 structure, which was not possible using dinucleotides. The idea of priming T7-mediated IVT with short oligonucleotides containing 3′-terminal guanosine was introduced even before the capping reagents and successfully used for incorporating the 2′-*O*-methylated or biotin-labeled nucleotides into the RNA 5′-end.^32^ Interestingly, the trinucleotide-based approach did not require methylation of the m^7^G ribose to prevent reverse incorporation thanks to an additional base pair between adenosine of the trinucleotide primer and thymidine at the −1 position of the template.^31^ A few years ago, several trinucleotide cap analogs became commercially available as CleanCap reagents,^33^ and a modified variant of a trinucleotide cap, m_2_^7,3′-*O*^GpppA_m_pG (Figure 3B), has been used for the production of the Comirnaty vaccine.^5^

Recently, we reevaluated the trinucleotide analogs of m^7^GpppA*pG and expanded this set with m^7^GpppNpG trinucleotides containing nucleobases other than adenosine (C, G, and U).^1^ The capping efficiencies during IVT on a template containing T7 ϕ6.5 promoter followed by a sequence of 35 nucleotides varied from 55–60% for pyrimidine analogs (N = C, C_m_, U, U_m_) through 80–85% for guanosine and *N*6-methyladenosine (N = G, G_m_, ^m6^A, ^m6^A_m_), to ca. 90% for adenosine (N = A, A_m_). The observed preference for trinucleotides containing purine nucleotides results from their ability to form an additional base pair with the template (Figure 2F).

In a follow-up study, we showed that tetranucleotide cap analogs m^7^GpppA_m_pG_m_pG are also efficient IVT primers (ca. 90% capping using ϕ6.5 promoter) and provide direct access to mRNAs with cap-2 structures.^34^ We then applied a similar approach to incorporate noncanonical caps including NAD, FAD, and UDP-sugars.^35^

Efficient, reproducible, and scalable synthesis of 5′-capped mRNA is crucial for therapeutic applications. The development of mRNA vaccines against SARS-CoV-2 has shown that both major approaches, namely, (i) post-transcriptional capping using the vaccinia system^6^ and (ii) co-transcriptional capping using trinucleotide cap analogs,^5^ are suitable for this purpose. The most evident difference between the two methods is access to mRNA with chemically modified caps. Although some GTP analogs are tolerated by the vaccinia enzyme as substrates for GMP transfer,^36^ the co-transcriptional capping with trinucleotides offers a more general platform for incorporating modifications. Those include natural methylations of 5′-terminal nucleotides (cap-1, cap-2, m^6^A_m_),^1,31,34^ noncanonical caps,^35^ synthetic modulators of translational properties,^37,38^ and functional groups for molecular labeling.^39−41^

## 4 Modification of 5′-Cap Structures

The exogenously delivered mRNA must effectively compete with endogenous mRNA for the translation machinery to elicit its therapeutic effect. The properties of mRNA that determine its competitiveness include the affinity for the translation machinery (especially translation initiation factor 4E, eIF4E), cellular stability, and immunogenicity. All of these are linked to the 5′-cap. Therefore, we have been looking for chemical modifications of the 5′-cap that may benefit mRNA-based therapeutics. The two most unique features of the 5′-cap, crucial for its specific interactions with cap-binding proteins (CBPs), are 7-methylguanosine and the 5′,5′-triphosphate bridge (Figure 1).

Initially, we realized that the 5′,5′-triphosphate bridge is particularly suitable for chemical modification since it binds tightly to various CBPs and is selectively hydrolyzed by Nudix-family enzymes (especially Dcp2), directing the mRNA to degradation. Among several phosphate modifications studied in the context of ARCA (m_2_^7,2′-*O*^GpppG),^42^ one appeared to be of particular interest: an *O*-to-*S* substitution within the β-phosphate. The modification creates an additional stereogenic center at the phosphorus, hence the so-called β-*S*-ARCA existed as a pair of diastereomers (Figure 3C), which exhibited slightly different biological properties.^43,44^ The D1 diastereomer bound to eIF4E with 4-fold higher affinity than the unmodified ARCA and RNAs capped with β-*S*-ARCA D1 were decapped by Dcp2 at a slower rate (Table 1). The D2 diastereomer also had a higher affinity for eIF4E (2-fold) and, when incorporated into RNA, prevented the decapping by Dcp2 and increased the mRNA half-life in cells.^43,45^ Hence, both compounds provided access to capped mRNAs with superior stability and translational activity. Particularly, the mRNAs capped with β-*S*-ARCA D1 produced almost 3-fold more protein in human immature dendritic cells than the corresponding ARCA-mRNA or mRNA capped post-transcriptionally.^45^ An antigen-encoding RNA containing β-*S*-ARCA D1 efficiently induced immune response, resulting in a 3-fold higher activation of antigen-specific T cells after intranodal RNA immunization of mice.^45^ This was a significant improvement in the emerging field of RNA vaccines, and β-*S*-ARCA D1 was used to cap mRNAs used in several clinical trials.^46,47^

**Table 1:** Biological Properties of Chemically Modified Cap Analogs and mRNAs Capped with Them

			translational activity		
cap analog	affinity for eIF4E, *K*_D_[nM]^a^	relative susceptibility to Dcp2^b^	rel transl efficiency	reference analog	conditions^c^
m_2_^7,2′-*O*^Gpp_S_pG D1 (β-*S*-ARCA D1)^43,45^	23.2 ± 0.8	↓	2.8 ± 0.3	m^7^GpppG	total firefly luciferase expression in HC11 cells; mRNAs isolated by RNeasy mini (QIAGEN) kit
			1.3 ± 0.3	m_2_^7,2′-*O*^GpppG	
			13.09 ± 0.31	m^7^GpppG	total firefly luciferase expression in iDCs; *mRNAs isolated by* MEGAclear (Ambion) kit
			2.74 ± 0.09	m_2_^7,2′-*O*^GpppG	
			3.88 ± 0.03	m^7^GpppG	total firefly luciferase expression in mDCs; *mRNAs isolated by* MEGAclear (Ambion) kit
			1.54 ± 0.02	m_2_^7,2′-*O*^GpppG	
m_2_^7,2′-*O*^Gpp_S_pG D2 (β-*S*-ARCA D2)^43,45^	51.8 ± 5.9	↓↓	5.1 ± 0.5	m^7^GpppG	total firefly luciferase expression in HC11 cells; mRNAs isolated by RNeasy mini (QIAGEN) kit
			2.4 ± 0.5	m_2_^7,2′-*O*^GpppG	
			6.57 ± 0.08	m^7^GpppG	total firefly luciferase expression in iDCs; mRNAs isolated by MEGAclear (Ambion) kit
			1.38 ± 0.03	m_2_^7,2′-*O*^GpppG	
			4.04 ± 0.05	m^7^GpppG	total firefly luciferase expression in mDCs; mRNAs isolated by MEGAclear (Ambion) kit
			1.60 ± 0.03	m_2_^7,2′-*O*^GpppG	
m_2_^7,2′-*O*^Gpp_BH3_pG D1^48^	25.4 ± 0.8	↓↓	2.25 ± 0.35	m_2_^7,3′-*O*^GpppG	total firefly luciferase expression in hiDCs; mRNAs isolated on Dynabeads MyOne (Invitrogen) magnetic beads
			1.03 ± 0.26	β-*S*-ARCA D1	
m_2_^7,2′-*O*^Gpp_BH3_pG D2^48^	75.8 ± 1.1	↓↓↓	1.66 ± 0.02	m_2_^7,3′-*O*^GpppG	
			1.04 ± 0.17	β-*S*-ARCA D2	
m_2_^7,2′-O^Gpp_S_p_S_G D1/D2 mix^49^	18.3 ± 0.9^d^	↓↓	1.60 ± 0.01	β-*S*-ARCA D1	total firefly luciferase expression in hiDCs; mRNAs isolated on Dynabeads MyOne (Invitrogen) magnetic beads
			2.50 ± 0.17	β-*S*-ARCA D2	
m_2_^7,2′-*O*^Gppp^5′-S^G (α-PSL)^2^	94.3 ± 7.1	—	2.75 ± 0.29	m^7^GpppG	total *Renilla* luciferase expression in HeLa cells; mRNAs isolated by NucleoSpin RNA Clean-up (MACHEREY-NAGEL) kit
			1.45 ± 0.36	m_2_^7,3′-*O*^GpppG	
			1.02 ± 0.22	β-*S*-ARCA D2	
m^7^Gppp-tr-pA_m_pG^51^	7.35 ± 0.71	↑	2.51 ± 0.18	m_2_^7,2′-*O*^GpppG	total *Gaussia* luciferase expression in JAWS II cells; uncapped RNA removed enzymatically, mRNAs purified by RP-HPLC
			0.99 ± 0.16	m^7^GpppA_m_pG	
Bn^7^G_m_pppG^53^	107.1 ± 4.7	n.d.	13.2 ± 2.7	m^7^GpppG	*Gaussia* luciferase activity in A549 cells 72 h post-transfection; mRNAs purified by RP-HPLC
			1.53 ± 0.41	m_2_^7,2′-*O*^GpppG	
bn^2^m^7^GpppG^54^	2.8-fold lower than m^7^GpppG^e^	n.d.	1.80 ± 0.34	m^7^GpppG	total firefly luciferase expression in HEK293 cells; mRNAs isolated by NucleoSpin RNA Clean-Up (MACHEREY-NAGEL) kit
			1.40 ± 0.59	m_2_^7,2′-*O*^GpppG	
m^7(LNA)^GpppA_m_pG^38^	n.d.	n.d.	∼5	m^7^GpppA_m_pG	total GFP expression in JAWS II cells 1 day post-transfection; mRNAs purified by RP-HPLC
			∼5	m_2_^7,2′-*O*^GpppG	

a Determined by fluorescence quenching titration (*K*_D_ for m_2_^7,2′-*O*^GpppG—92.6 ± 2.6; for m^7^GpppA_m_pG—33.8 ± 2.6).

b Decapping rate relative to ARCA-RNA.

c Data from different experimental setups should not be compared directly.

d Value for m^7^Gpp_S_p_S_G D1.

e Determined by DSF; n.d.—no data.

Recently, we gained a deeper insight into the molecular basis of the beneficial “thio effect” in β-*S*-ARCA by co-crystallization of their complexes with eIF4E.^37^ We found that the key driving force for complex stabilization is an electrostatic interaction between the negatively charged sulfur atom and positively charged Arg and Lys residues in the protein binding site (Figure 3G). We believe that a similar mechanism underlies the properties of our boranophosphate and dithiodiphosphate cap analogs (Table 1).^48,49^

Despite the promising properties of β-*S*-ARCA and structurally related analogs, their chemical synthesis and isolation in a diastereomerically pure state, especially in bulk, has been a challenge. This problem has recently been addressed by the 5′-phosphorothiolate modification of the guanosine portion of the ARCA structure (termed α-PSL cap, Figure 3E), which contains an *O*-to-*S* substitution but does not create a stereogenic center.^2^ Although it does not significantly stabilize the complex with eIF4E nor prevent decapping by Dcp2, mRNAs capped with the α-PSL analogue are translated in HeLa cells comparably to mRNAs capped with β-*S*-ARCA D2 (Table 1).

We also explored the concept of mimicking the phosphate residues with a triazole moiety, which had been shown to be biocompatible with many DNA-related processes.^50^ Incorporating the triazole into the 5′,5′-oligophosphate chain enables the assembly of cap structures via click chemistry. From dozens of phosphotriazole dinucleotide analogs synthesized by Cu(I)-catalyzed azide–alkyne cycloaddition (CuAAC), we were able to select several that provided RNAs with translational properties similar to those of ARCA-capped ones.^3^ In the follow-up studies, we combined these modifications with a trinucleotide approach to improve capping efficiency and enable the synthesis of cap-1 analogs.^51^ One of the compounds (Figure 3F), showed translational activity in cells comparable to the natural cap-1 structure (Table 1), making it a promising candidate for further optimization and paving the way for alternative capping strategies using click chemistry.

Other chemical modifications of 5′-cap structure investigated by us and others focus on the 7-methylguanosine portion. Substitution of the *N*7-methyl with benzyl derivatives stabilizes the interaction with eIF4E, making them promising candidates for translation inhibitors,^52^ and in some cases has a moderately positive effect on translation efficiency when incorporated into mRNA (Table 1).^53^ Similar modifications of the *N*2 position of m^7^G result in up to 2-fold increased expression in HEK293 cells (Table 1).^54^ Another example is a trinucleotide cap-1 structure with LNA modification of m^7^G (Figure 3D), which is not as good as an IVT primer as m^7^GpppA_m_pG, but the resulting RNA yields 5-fold more protein than mRNA with unmodified cap-1 (Table 1).^38^

Recent reports on the reversible nature of the *N*6 methylation of cap-adjacent adenosine (Figure 1B) invite investigation of synthetic modifications at this position.^55^ Such modified mRNA was prepared by chemoenzymatic alkylation of capped RNA using Pcif1 methyltransferase and a propargyl-AdoMet analog.^56^ Its translation in HEK-NF-κB cells yielded 2-fold less reporter protein than that observed for cap-1 RNA, and the expression of *N*6-methylated cap-1-RNA was even lower. The novel chemoenzymatic and tri(tetra)nucleotide capping technologies will surely enable broader exploration of the chemical space around the mRNA 5′-cap.

## 5 Biological Function and Emerging Potential of Poly(A) Modifications

**Figure 4:**
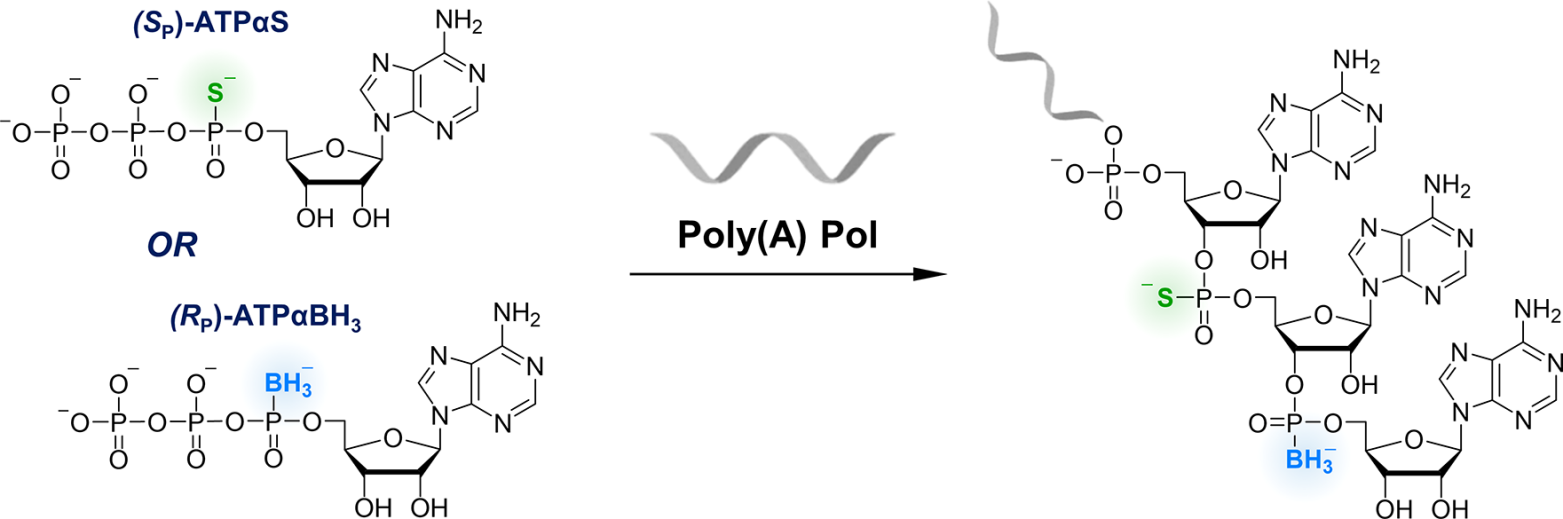
Multiple modifications of poly(A) tail using phosphate-modified ATP analogs and PAP.^59^

The modification of the 3′-end also offers potential benefits for mRNA therapeutics. Poly(A) is added during the nuclear processing of pre-mRNA and facilitates mRNA export to the cytoplasm. In the cytoplasm, the mRNA poly(A) tail associates with poly(A)-binding protein (PABP) protein, which promotes translation as a part of the translation initiation complex and stabilizes poly(A) by protecting it from deadenylases. Poly(A) shortening is the first step preceding mRNA degradation in both the 3′-to-5′ and 5′-to-3′ directions.^57^ As such, poly(A), similar to the 5′-cap, is essential for both mRNA translation and stability, but in contrast to 5′-cap, poly(A) tail modifications have only recently come under investigation in the context of increasing mRNA translational potential or stability. Poly(A) can be directly encoded in the DNA template and thereby incorporated into mRNA during in vitro transcription or added post-transcriptionally with the use of poly(A) polymerases (PAPs). The first approach is more straightforward, but the instability of long adenine stretches during DNA plasmid amplification poses a challenge. Sequence engineering is one solution to modify the stability of DNA plasmids and potentially also increase the stability of poly(A) tails in mRNA.^58^ The poly(A) tail can also be added post-transcriptionally using template-independent PAPs that utilize ATP as a substrate. Chemical poly(A) modifications can be incorporated by replacing or mixing ATP with an ATP analogue that acts as a substrate. If the resulting modified poly(A) fragment can be further extended by PAP, multiple modifications of poly(A) are possible. We have shown that (*S*_*P*_)-ATPαS, added into IVT mix along with ATP can be incorporated into poly(A) either by T7 or PAP polymerase producing phosphorothioate-modified mRNA (Figure 4).^59^ The use of T7 polymerase and ATP/(*S*_*P*_)-ATPαS mixtures at different ratios resulted in mRNAs that were modified both in the poly(A) tail and in the rest of the mRNA body. Such mRNAs had low translational activity, likely because phosphorothioate modifications in the coding sequence interfere with translation. In contrast, the use of bacterial PAP and ATP/(*S*_*P*_)-ATPαS mixtures afforded mRNAs modified exclusively in the poly(A). Such mRNAs had generally lower susceptibility to deadenylation in vitro but neither significantly reduced nor increased translational activity in HeLa cells. The incorporation of a corresponding boranophosphate analogue, (*R*_*P*_)-ATPαBH_3_ (Figure 4), resulted in mRNAs with decreased translational potential. Overall, the study showed that the application of PAP and ATP analogs can be applied to modify poly(A) tails of mRNA, but more work is necessary to enable modifications in a more controlled way and identify patterns that increase the protein output.

Others focused on modifications of the 3′-terminal part of poly(A). 3′-Azido-2′3′-dideoxyATP and 2′-azido-2′deoxyATP and yeast poly(A) polymerase (PAP) were used to add azido residues to the 3′-end of polyadenylated mRNA.^60^ The polymerase incorporated, respectively, single or multiple (2–6 by average) modified AMP residues into the poly(A). The mRNAs modified with multiple azido moieties had increased translational activity, and the effect was more pronounced after subsequent fluorescent labeling. We also explored the direct chemical and chemoenzymatic modification of the mRNA 3′-end in the context of fluorescent labeling, which is discussed in the next section.

## 6 mRNA 5′- and 3′-End Labeling for Visualization and Localization of RNA in Cells

Labeling and visualizing mRNA molecules in cells play crucial roles in understanding their function and dynamics. To provide molecular tools suitable for the investigation of dynamic cellular processes involving mRNA ends, we focused on labeling the 5′-cap and the 3′-terminus. The labeling of the mRNA ends has the advantage of being site-specific and sequence-independent, making it predictable and applicable to any IVT mRNA.

**Figure 5:**
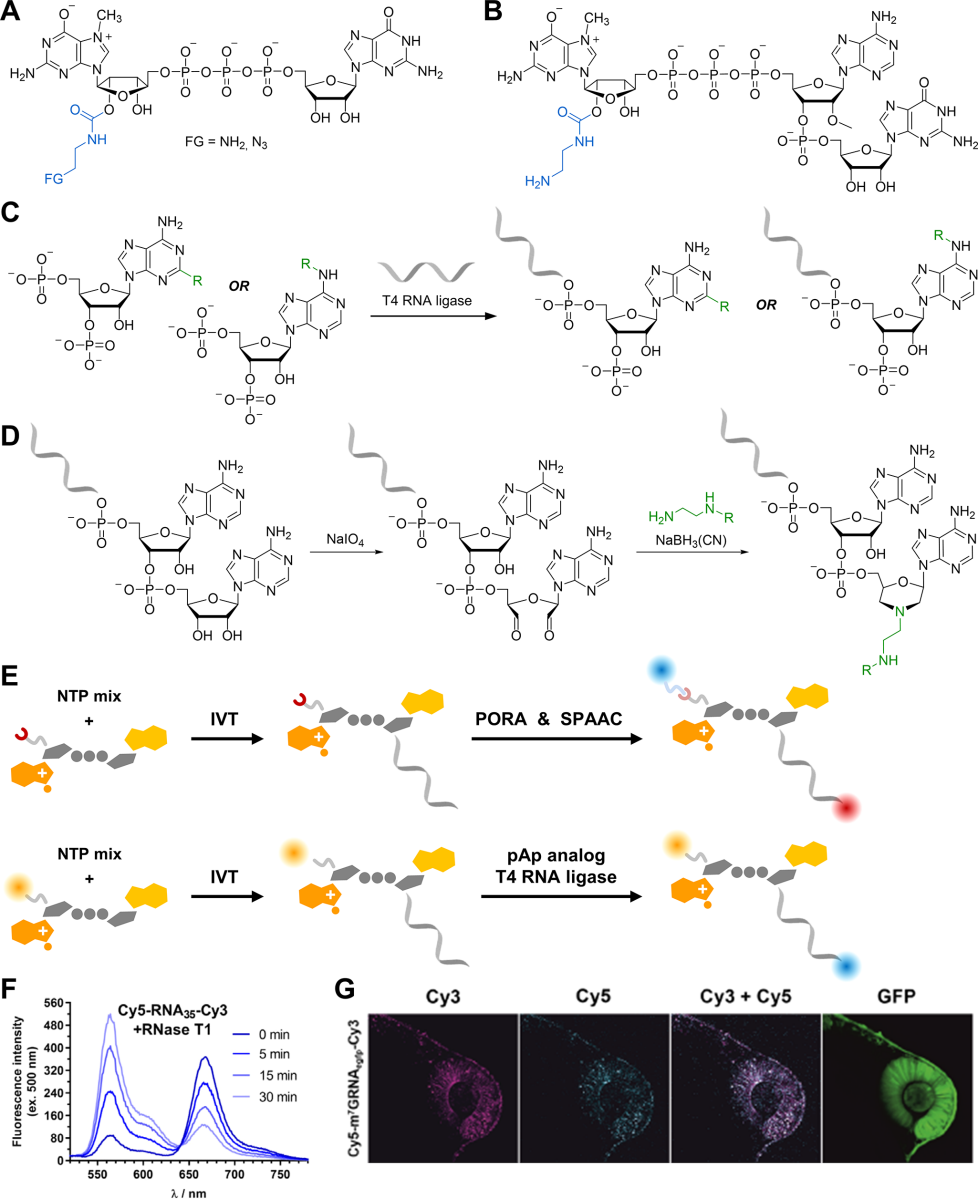
Labeling of mRNA ends: (A) dinucleotide reagents and (B) trinucleotide reagents for co-transcriptional labeling of mRNA 5′-end; (C) chemical modification of RNA 3′-end by PORA; (D) chemoenzymatic modification of RNA 3′-end using pAp analogs and T4 RNA ligase; (E) synthesis of dually labeled mRNAs; (F) dual-labeled RNA probes exhibit FRET and allow monitoring of enzymatic decay as time-dependent changes in emission spectra after addition of RNase T1 to Cy5-RNA35-Cy3; (G) Visualization of dual-labeled GFP mRNA in zebrafish embryo. Panels F and G reproduced with permission from ref (4). Copyright 2022 Oxford University Press.

The proper label placement within the 5′-cap is crucial, as the modification can easily interfere with mRNA synthesis by IVT or with mRNA translatability. Based on the crystal structures of cap–eIF4E complexes, we and others designed cap analogs with tags attached directly to the solvent-exposed 2′/3′-position of the m^7^G ribose.^61−63^ Such modifications are well tolerated by eIF4E, and thus, such capped RNAs are quite efficiently translated. The substituent also prevents the cap from incorrect incorporation during IVT (Figure 2D). However, the limited range of tags that are incorporable this way encouraged us to incorporate spacers of various lengths, terminated with an amine group suitable for conjugation with *N*-hydroxysuccinimide (NHS) esters (Figure 5A)^64^ or with an azido-modified group enabling the bioorthogonal labeling of the 5′-end of in vitro transcribed mRNAs.^65^ Recently, we combined this functionalization strategy with a trinucleotide-based priming approach (Figure 2E, Figure 5B), which significantly increased the co-transcriptional capping efficiency and allowed the production of mRNAs with cap-1 structure.^40^

The 3′-end of RNA can be labeled by ligating with a pNp analog substituted within the terminal phosphate or the nucleobase (Figure 5C).^40,66^ We have designed a pAp analog suitable for the efficient labeling of full-length mRNAs.^40^ Although this labeling method is robust and versatile, the resulting mRNAs contain a 3′-phosphate moiety that may alter their biological properties.

From a chemical point of view, the feature that distinguishes the 3′-terminal nucleotide from internal nucleotides is the presence of a *cis*-diol. *cis*-Diol can undergo selective periodate-mediated oxidation followed by reductive amination (PORA), resulting in the conversion of the 3′-terminal ribose to a morpholine derivative (Figure 5D). Recently, we discovered that ethylenediamine derivatives exhibit exceptional reactivity during the reductive amination step, which resulted in an improved protocol for the direct chemical labeling of the mRNA 3′-end.^4^ Importantly, both the chemical modification and the labeling procedure had no effect on protein output.

Finally, to provide access to dual-labeled mRNAs, we successfully combined either enzymatic ligation with pAp analogs or the optimized PORA protocol with co-transcriptional functionalization of the 5′-cap (Figure 5E).^4,40^ The introduction of a pair of tracers at both ends of the mRNA expands possibilities for studying cellular processes. Such probes containing FRET pair fluorophores have proven useful for investigating the distance between the 5′ and 3′-ends of mRNA,^66^ studying mRNA decapping with in vitro reconstituted molecular condensates,^41^ and visualizing mRNA localization and expression in vivo (Figure 5F).^4^

## 7 Harnessing Modifications to Facilitate mRNA Purification and Improve Quality

**Figure 6:**
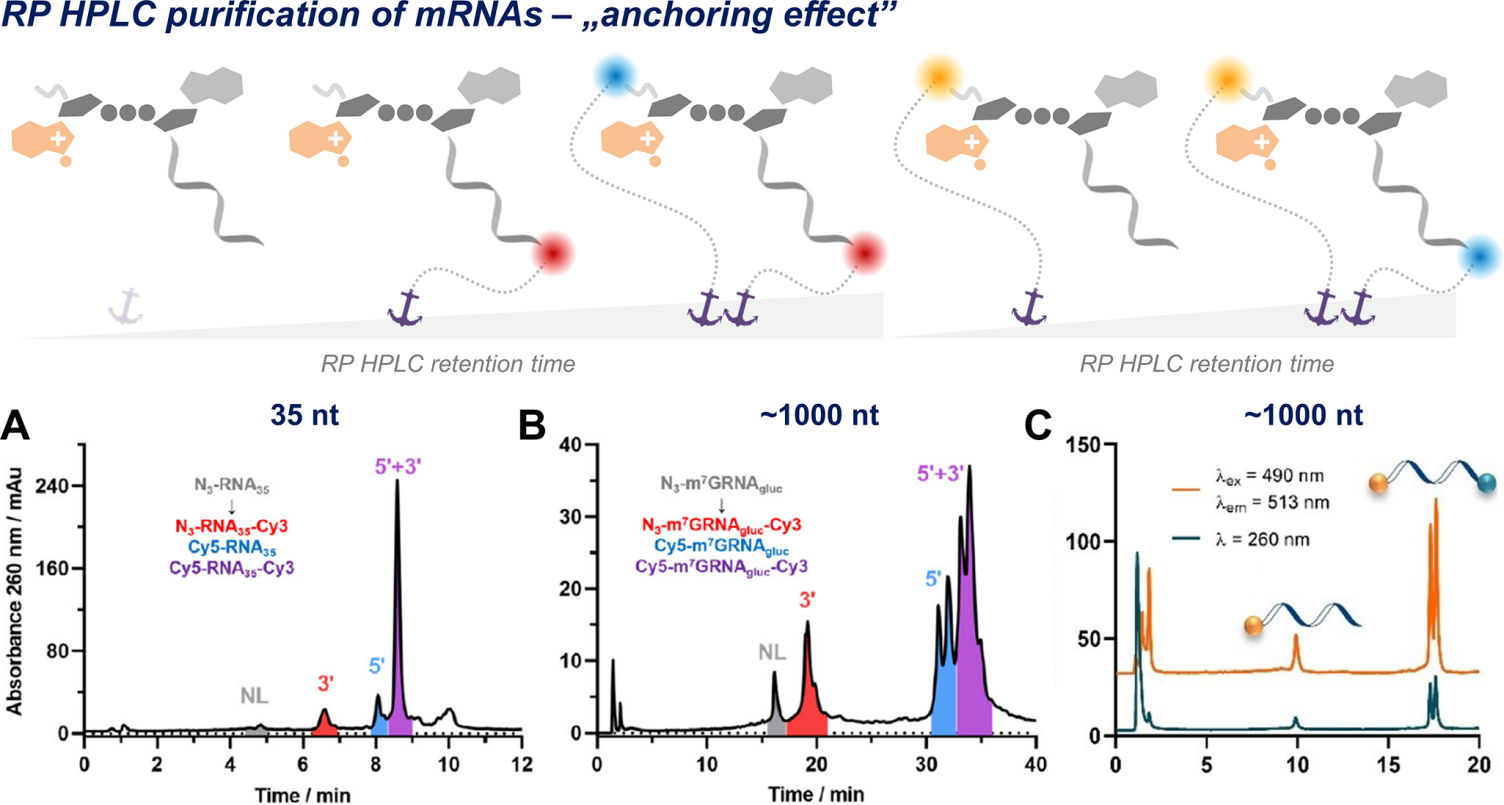
Harnessing the anchoring effect of hydrophobic fluorescent tags for RNA purification by RP HPLC. (A) Purification of short (35 nt) RNA probe labeled with Cy3 and Cy5. (B) Purification of *Gaussia* luciferase mRNA labeled with Cy3 and Cy5. (C) Purification of mRNA labeled with FAM and Cy5. NL, not labeled; 3′, RNA labeled at the 3′-end; 5′, RNA labeled at the 5′-end; 5′+3′, RNA labeled at both ends. Doubling of peaks is sometimes observed due to the presence of isomeric forms of labeled RNAs. Panels A and B reproduced with permission from ref (4). Copyright 2022 Oxford University Press. Panel C reproduced with permission from ref (40). Copyright 2021 John Wiley and Sons.

Reversed-phase chromatography (RP-HPLC) is one of the methods enabling effective mRNA purification, including removal of reactogenic double-stranded impurities.^1,67,68^ The hydrophobic labels incorporated into mRNA using synthetic capping reagents or by modification of poly(A) may alter the physicochemical properties of RNA significantly, opening up opportunities for facilitated isolation/purification by HPLC. The use of hydrophobic tags to purify short-capped RNA sequences has been proposed in the past.^69^ Surprisingly, we have observed that even for very long RNAs, the incorporation of a fluorescently labeled 5′-cap or fluorescent modification of the poly(A) tail remarkably extends their retention time on the RP-HPLC column. The magnitude of the effect depends on the number of labels and their hydrophobicity, which enables not only the removal of uncapped/unlabeled mRNA species but also an effective separation of monolabeled mRNAs from dual-labeled ones or even isomeric forms of the labeled species (Figure 6). This hydrophobic effect, i.e., slowing mRNA migration by the presence of a hydrophobic moiety acting like an anchor, gives unprecedented access to highly homogeneous dual-labeled mRNA probes.^4,40^ Recently, the hydrophobic effect for mRNA was combined with photocleavable tags to facilitate the purification of unmodified capped mRNAs.^70^

## 8 Future Prospects and Challenges

mRNA technology has had a tremendous impact on how the world works over the past three years. The first two COVID-19 vaccines were developed, approved, and marketed in record time and administered in an unprecedented number of doses. They were also the first therapeutic products approved for sale based on mRNA technology. Undoubtedly, the potential of mRNA technology is much greater, as it makes possible delivery of a recipe for any protein that will be produced in the patient’s body according to the natural mechanism of protein biosynthesis, which opens treatment possibilities limited only by human imagination. The best evidence for this is the hundreds of clinical trials that have been initiated for mRNA-based therapies to meet various medical needs. mRNA technology not only gives hope for more prophylactic vaccines (currently under development are vaccines against influenza, HIV, RSV, and Zika virus, among others) but also promises therapeutic cancer vaccines, including personalized ones, that are designed to destroy cancer cells of a patient using their own immune system. mRNA technology is also tested in clinical trials for rare genetic and metabolic diseases, which have genesis in the abnormal production of certain proteins in the body. Clinical trials verifying such therapies include diseases such as phenylketonuria, cystic fibrosis, hemophilia, and more. Other applications of mRNA include regenerative medicine, cellular therapies, or delivering enzymes for precise genome editing (e.g., CRISPR-Cas9). The success of anti-COVID vaccines and the enormous potential of mRNA in other therapeutic areas have led to tremendous interest in this technology from the pharmaceutical industry, businesses, and the general public.

However, to make the expansion beyond antiviral vaccines possible, further development of mRNA platforms is necessary. Creating therapeutic mRNAs that will undergo even more efficient and sustained expression and can be administered repeatedly without triggering the immune system is a challenge for the future, which may be addressed with the use of chemical methods and tools, including modifications of the mRNA ends. Another issue for the research community is establishing standards related to mRNA production, purification, quality control, and biochemical evaluation. The methods significantly evolved in recent years, and it is increasingly better understood that the results of cell culture and in vivo assays may significantly depend on the purification standards (especially double-stranded mRNA content) and the type of targeted cells/biological setup.^1,71^ There appears to be also room for further improvement of translational efficiency, but to do so, we need to better understand the cellular metabolism of mRNA, including the role of post-transcriptional modifications, and hopefully identify new mechanisms that modulate the expression of therapeutic mRNA. To treat genetic diseases, it will be necessary to invent new approaches that increase mRNA durability in vivo. To that end, the poly(A) tail and mRNA circularization by chemical methods offer a fantastic playground for chemists. Despite the success of LNPs as a method for delivering mRNA vaccines, efficient and selective delivery of mRNA to individual tissues or cell types remains largely unaddressed. Selective delivery of mRNAs to particular tissues would allow for investigating tissue-specific solutions at the level of mRNA sequences and structural modifications including the cap and poly(A). One thing is certain, the next decade will be marked by therapeutic mRNA: how much will be achieved to a large extent depends on the effectiveness and inventiveness of the research community in the search for new solutions and improvements to this technology.
